# In silico prediction of deleterious non-synonymous SNPs in *STAT3*

**DOI:** 10.2478/abm-2023-0059

**Published:** 2023-10-18

**Authors:** Athira Ajith, Usha Subbiah

**Affiliations:** Human Genetics Research Centre, Sree Balaji Dental College and Hospital, Bharath Institute of Higher Education and Research, Chennai 600100, Tamil Nadu, India

**Keywords:** deleterious, In silico, prediction, single nucleotide polymorphism, *STAT3*

## Abstract

**Background:**

*STAT3*, a pleiotropic transcription factor, plays a critical role in the pathogenesis of autoimmunity, cancer, and many aspects of the immune system, as well as having a link with inflammatory bowel disease. Changes caused by non-synonymous single nucleotide polymorphisms (nsSNPs) have the potential to damage the protein's structure and function.

**Objective:**

We identified disease susceptible single nucleotide polymorphisms (SNPs) in *STAT3* and predicted structural changes associated with mutants that disrupt normal protein–protein interactions using different computational algorithms.

**Methods:**

Several *in silico* tools, such as SIFT, PolyPhen v2, PROVEAN, PhD-SNP, and SNPs&GO, were used to determine nsSNPs of the *STAT3*. Further, the potentially deleterious SNPs were evaluated using I-Mutant, ConSurf, and other computational tools like DynaMut for structural prediction.

**Result:**

417 nsSNPs of *STAT3* were identified, 6 of which are considered deleterious by *in silico* SNP prediction algorithms. Amino acid changes in V507F, R335W, E415K, K591M, F561Y, and Q32K were identified as the most deleterious nsSNPs based on the conservation profile, structural conformation, relative solvent accessibility, secondary structure prediction, and protein–protein interaction tools.

**Conclusion:**

The in silico prediction analysis could be beneficial as a diagnostic tool for both genetic counseling and mutation confirmation. The 6 deleterious nsSNPs of *STAT3* may serve as potential targets for different proteomic studies, large population–based studies, diagnoses, and therapeutic interventions.

Signal transducer and activator of transcription factors (STAT) regulate key aspects of cell growth, survival, and differentiation. STAT pathway is linked to the Janus kinases (JAK) family of proteins, i.e., STATs are activated by phosphorylation by JAKs. STAT1, STAT2, STAT3, STAT4, STAT5a, STAT5b, and STAT6 are members of the mammalian STAT family [1]. Each member responds to different kinds of cytokines and growth factors and has a distinct role in signal transduction. When certain chemical signals activate STAT proteins, they move into the nucleus of the cell and bind to specific areas of DNA. STAT3 is found in all of the body's tissues and plays a significant role in the development and function of various physiological systems. The protein is involved in the regulation of inflammation, which is one of the ways the immune system responds to infection or injury. STAT3 activation occurs as a result of cytokine binding and is required for cellular responses via JAK1, JAK2, or TYK2. The non-classical functions of STAT3 include the induction of microRNAs, binding to non-canonical motifs, formation of more complex signaling cascades such as STAT tetramers, and contributing to epigenetic remodeling [2].

The major disorders associated with *STAT3* include hyper-IgE recurrent infection syndrome 1, autosomal dominant and autoimmune disease, multisystem, and infantile-onset. Genome-wide association studies (GWAS) have revealed the role of *STAT3* in diseases like mean corpuscular volume, multiple sclerosis, inflammatory bowel disease, Crohn disease, ankylosing spondylitis, psoriasis, ulcerative colitis, and sclerosing cholangitis. Constitutive activation and regulation of *STAT3* induce cellular transformation and can exert various types of tumors including head and neck, prostate, breast, and leukemia [3].

*STAT3* can enhance cancer stem cell self-renewal and differentiation by altering the gene expression via the epithelial–mesenchymal transition (EMT) [4]. *STAT3* induces VEGF expression in the association of hypoxia-inducible factor 1-alpha (HIF1A) to promote tumor angiogenesis [5]. An examination of the literature reveals that *STAT3* has been found to be significantly associated with autoimmune thyroid diseases (AITDs) and further suggests that this may alter the level of thyroid autoantibodies in AITD patients [6]. The rs744166 polymorphism in *STAT3* has been associated with a lower risk of gastric cancer in the Chinese population [7].

Single nucleotide polymorphism (SNP) is considered for over 90% of sequence variations in the human genome [8] and plays an important role in investigating potential biomarkers and identifying common genetic variants. These SNPs may have a deleterious or neutral effect on protein function associated with a variety of diseases and disorders. Missense variants, by substituting amino acids, cause alterations in protein-coding regions [9]. In silico methods have been developed for screening functional SNPs, detecting the effect of damaging non-synonymous single nucleotide polymorphisms (nsSNPs) in selected proteins, and predicting structural changes based on single amino acid substitution in the protein. It is a time and cost–effective alternative to experimental techniques and has already incorporates the process of screening for deleterious nsSNPs, and thus could be used in future studies.

In this study, detailed investigations have been carried out using several in silico tools to evaluate the potentially detrimental effects on nsSNPs of the *STAT3* gene and to identify their structural and functional impact on the STAT3 protein.

## Methods

Various in silico tools were used to predict the variations in the structure, stability, and function of the *STAT3* gene.

### Retrieving nsSNPs

The nsSNP distribution of the *STAT3* gene (Accession: NP_003141.2) was collected from the National Center for Biotechnology Information (NCBI) dbSNP database (https://www.ncbi.nlm.nih.gov/projects/SNP) and the protein sequence was retrieved from the UniProt database (UniProtKB – P40763 (*STAT3*_HUMAN)).

### Prediction and identification of deleterious SNPs

To estimate the functional repercussions of nsSNPs in the coding area acquired from the dbSNP database, 7 best-performing web tools were employed sequentially.

Sorting Intolerant From Tolerant (SIFT; https://sift.bii.a-star.edu.sg/) [10] predicts the amino acid substitution effects and the damaging effect on protein function based on homology to identify the tolerated and deleterious SNPs. The SIFT probability score ≤0.05 indicates deleterious and those >0.05 indicate tolerated. The rsIDs of nsSNPs acquired from NCBI's dbSNP database were submitted as input queries.

Polymorphism Phenotyping v2 (PolyPhen2; http://genetics.bwh.harvard.edu/pph2/) [11] uses the protein sequence and amino acid substitutions in the sequence to predict the structural and functional effects on the protein. The output of the prediction shows the nsSNPs as “PROBABLY DAMAGING” with a score of 0.7–1, “POSSIBLY DAMAGING” with a score of 0.5–0.8, or “BENIGN”.

Based on the alignment score of a protein, Protein Variation Effect Analyzer (PROVEAN; http://provean.jcvi.org/index.php) [12] analyzes the functional effect of amino acid substitutions in the protein. The SNPs with a PROVEAN score of ≤ −2.5 are considered to have a damaging effect and > −2.5 indicates a neutral effect on the protein.

Based on evolutionary relationships, molecular activities, and interactions with other proteins, Protein Analysis through Evolutionary Relationship (PANTHER; https://www.pantherdb.org/tools) [13] predicts that a specific nsSNP will have a functional impact on the protein using position-specific evolutionary conservation scores. The required input query for this prediction was protein sequence and amino acid variants.

SNPs&GO (http://snps.biofold.org/snps-and-go/snps-and-go.html) [14] used the corresponding protein query sequence to predict the use of a reliability index (RI) that would enable the ascertainment of disease-relevant mutations in a protein sequence.

Predictor of human Deleterious Single Nucleotide Polymorphisms (PhD-SNP; http://snps.biofold.org/phd-snp/phdsnp.html) [15] is an online predictor based on the support vector machine (SVM), and is used to classify the nsSNPs as disease-related or neutral polymorphism.

### The impact of nsSNPs in determining the structural stability of proteins

I-Mutant 3.0 (http://gpcr2.biocomp.unibo.it/cgi/predictors/I-Mutant3.0/I-Mutant3.0.cgi) [16] was used to evaluate changes in protein stability due to nsSNPs based on both protein sequence and protein structure. The stability changes and related ΔΔG value were calculated using I-Mutant 3.0. ΔΔG value is the difference in the Gibbs free energy of the mutated protein and the wild-type [17]. A ΔΔG value <0 indicates that the variation has a negative impact on protein stability. A ΔΔG value >0, on the other hand, suggests that the variation improves protein stability.

### Phylogenetic conservation analysis of nsSNPs

The ConSurf web server (http://consurf.tau.ac.il) [18] analyzes the evolutionary rate of conservation of the amino acids in the protein sequence. The input was the FASTA format of protein sequence, which calculates conservation scores in the range of 1–9. A score range of 1–4 indicates variable, a 5–6 score indicates intermediate, and a 7–9 score is a conserved one.

### Relative solvent accessibility prediction

NetsurfP-2.0 [19] predicts the accessibility of solvents, secondary structure, disorders, and phi/psi dihedral angles of amino acids in an amino acid sequence. The FASTA format of the STAT3 protein sequence was submitted as an input query for this server.

### Prediction of structural effects of nsSNPs upon mutation

HOPE (http://www.cmbi.ru.nl/hope/) [20] was used to investigate the impact of point mutations on the protein structure. A protein sequence or an accession code for the protein of interest was used as the input query. The structural data were gathered from the distributed annotation system (DAS) servers as well as the Uniprot database. HOPE also provides information on structural differences between mutant and normal residues.

### Prediction of protein secondary structure and analyses of protein–protein interactions

SOPMA (https://npsa-prabi.ibcp.fr/cgibin/npsa_automat.pl?page=npsa_sopma.html) [21] was used to predict the secondary structure of STAT3 protein using 5 algorithms. The FASTA sequence of the protein was given as input for SOPMA prediction.

The Search Tool for the Retrieval of Interacting Genes/Proteins (STRING; https://string-db.org/) database predicts the functional linkages between proteins as well as their associations by combining data from physical interactions and curated biological pathway databases.

### Predicting ligand binding site effects of nsSNPs

Raptor X gives the prediction of the secondary and tertiary structure of the protein, contact and distance maps, solvent accessibility, disordered regions, functional annotation, and binding sites based on a 3D model. Binding site prediction is calculated by the pocket multiplicity, which determines the quality of the projected pocket. The predicted pocket is more accurate when the score is greater than 40.

### Analysis of gene–gene interactions

GeneMANIA [22] (https://genemania.org/) predicts the gene–gene interaction using a large set of functional association data including protein and genetic relationships, pathways, co-expression, co-localization, and protein domain similarity. *STAT3* was the input query entered into the software.

### Structural analysis of *STAT3* protein

AlphaFold (https://alphafold.ebi.ac.uk/) is the most accurate technique for predicting protein structure by neural network–based model. The AlphaFold model includes the coordinates of every heavy atom in a protein as well as “its confidence in the form of a projected LDDT-C score (pLDDT) per residue” [23]. The LDDT score, which ranges from 0 to 100, describes how closely the protein model resembles the reference structure without superposition [24]. The Uniprot accession code of STAT3 was used to acquire the wild-type protein structures from the AlphaFold Protein Structure Database.

### Analysis of protein stability

The impact of a single point mutation on the stability, conformation, flexibility of proteins, and the visualization of protein dynamics, were evaluated using the DynaMut server (http://biosig.unimelb.edu.au/dynamut/). DynaMut employs normal mode analysis (NMA) to compare the free energy change (ΔΔG) between the wild-type and mutant structures. DynaMut additionally provides structure-based predictions for mCSM [25], SDM [26], and DUET [27], as well as the ΔΔG prediction of an elastic network contact model (ENCoM) based on NMA. Moreover, DynaMut uses ENCoM-based difference in vibrational entropy (ΔΔSVib) to determine whether the mutation will be more or less flexible. The wild-type structure in PDB format and the variant amino acid were given as input.

## Result

All the SNP data for the *STAT3* gene were retrieved from the NCBI dbSNP database, comprising a total of 26,861 SNPs. Among the reported SNPs, 24,378 were introns, 417 nsSNPs (missense), 307 synonymous, 3 inframe deletions, and 2 inframe insertions. We exclusively chose nsSNPs for further analysis in this study as they change the encoded amino acid.

### Prediction of functional nsSNPs in *STAT3*

Various in silico prediction tools such as SIFT, Polyphen-2, PROVEAN, SNP & GO, PANTHER, and PhD–SNP were used to analyze disease-associated SNPs. Initially, all the 417 nsSNPs were loaded to the SIFT server, which predicted 160 nsSNPs as tolerated or deleterious, whereas the remaining SNPs were not found. Out of this, 67 nsSNPs were classified as deleterious with a SIFT score ≤0.05 and the remaining were tolerated. The nsSNPs were then submitted for PolyPhen2 analysis. To increase the accuracy of the prediction, the combined prediction of both SIFT and PolyPhen, such as the nsSNPs with SIFT score ≤0.05 and PolyPhen score >0.90, was selected and 9 nsSNPs were identified as deleterious. The selected 9 nsSNPs were subjected to other in silico tools, namely PROVEAN, SNP & GO, PANTHER, and PhD–SNP, and the results are given in **Table 1**.

**Table 1.** List of nsSNPs of *STAT3* gene predicted as deleterious by different bioinformatics tools

**S. No.**	**rs ID**	**Alleles**	**Amino acid change**	**SIFT (score)**	**Polyphen (Humvar) (score)**	**PANTHER**	**SNP & GO (RI)**	**PROVEAN (score)**	**PHD -SNP (RI)**	**MUTANT I DDG value (<0)**
1	rs145786768	C/A	V507F	Deleterious (0.004)	Probably damaging (0.990)	Probably damaging	Disease (9)	Deleterious (−3.744)	Disease (6)	Decrease (−2.49)
2	rs193922716	G/A	R335W	Deleterious (0)	Probably damaging (0.996)	Probably damaging	Disease (3)	Deleterious (−5.816)	Neutral (1)	Decrease (−0.36)
3	rs193922717	C/T	E415K	Deleterious (0.003)	Probably damaging (0.955)	Probably damaging	Disease (5)	Deleterious (−3.097)	Neutral (0)	Decrease (−1.00)
4	rs193922719	T/A	K591M	Deleterious (0.002)	Possibly damaging (0.751)	Probably damaging	Disease (8)	Deleterious (−4.949)	Disease (6)	Decrease (−0.13)
5	rs1803125	G/T	Q32K	Deleterious (0.025)	Possibly damaging (0.868)	Probably damaging	Disease (0)	Neutral (−1.975)	Disease (3)	Decrease (−0.41)
6	rs11547455	G/A	S629F	Deleterious (0.001)	Possibly damaging (0.481)	Probably damaging	Disease (5)	Deleterious (−3.097)	Disease (1)	Increase (0.64)
7	rs11547455	G/A	S727F	Deleterious (0.002)	Probably damaging (0.974)	Probably damaging	Neutral (0)	Deleterious (−3.858)	Neutral (1)	Decrease (−0.20)
8	rs374063766	C/G	Q198H	Deleterious (0.035)	Probably damaging (0.965)	Probably damaging	Neutral (3)	Neutral (−1.942)	Neutral (3)	Decrease (−0.82)
9	rs11547455	G/A	S727F	Deleterious (0.002)	Probably damaging (0.974)	Probably damaging	Neutral (0)	Deleterious (−3.858)	Neutral (1)	Decrease (−0.20)

nsSNPs, non-synonymous single nucleotide polymorphisms; PANTHER, Protein Analysis Through Evolutionary Relationship; PhD-SNP, Predictor of human Deleterious Single Nucleotide Polymorphisms; RI, reliability index; SNP, single nucleotide polymorphism.

According to PROVEAN results, 6 nsSNPs were predicted as disease-causing and 3 were neutral. Through the PANTHER tool, 6 nsSNPs were predicted as probably damaging, 1 was possibly damaging, and 1 was found to be benign. Moreover, SNP & GO predicted 6 of these 9 nsSNPs as disease-relevant mutations in *STAT3* with a RI. Finally, 6 nsSNPs were determined as deleterious by all the above online software. rs145786768, rs193922716, rs193922717, rs193922719, rs1064116, and rs1803125 with a change in amino acid V507F, R335W, E415K, K591M, F561Y, and Q32K were picked as the most deleterious nsSNPs since they showed a mutation in a majority of the in silico prediction tools. In addition, we used the I-Mutant server to analyze the effects of nsSNPs on protein stability. The result showed that 6 nsSNPs have a ΔΔG value < −0.5, which indicates decreasing stability of the STAT3 protein (**Table 1**).

### Conservation profile and structural conformation of nsSNPs in *STAT3*

**Figure 1.**
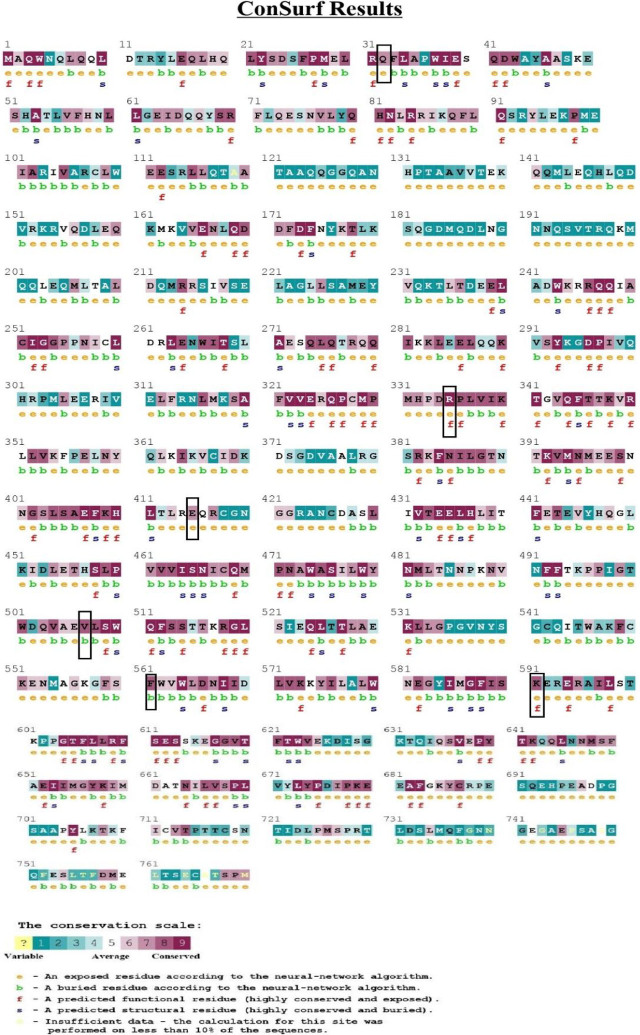
Evolutionary conservancy of STAT3 by ConSurf server. The high-risk nsSNPs are denoted by black boxes. nsSNPs, non-synonymous single nucleotide polymorphisms; STAT, signal transducer and activator of transcription factors.

Through the ConSurf web server, the evolutionary conservancy of 6 nsSNPs of STAT3 protein was analyzed, together with the identification of putative, structural, and functional residues. The results showed that all the 6 nsSNPS, namely V507F, R335W, E415K, K591M, F561Y, and Q32K, were highly conserved and the variants are indicated in black boxes in **Figure 1**. The result of ConSurf is shown in **Table 2**.

**Table 2.** Analysis of evolutionary conservation profile of high-risk nsSNPs of STAT3 by ConSurf

**Amino acid change**	**Conservation score**	**Buried/exposed**	**Functional/structural**
V507F	8	Buried	-
R335W	9	Exposed	Functional
E415K	8	Exposed	-
K591M	9	Exposed	Functional
F561Y	8	Exposed	Functional
Q32K	7	Exposed	-

nsSNPs, non-synonymous single nucleotide polymorphisms; STAT, signal transducer and activator of transcription factors.

### Relative solvent accessibility prediction

The variants with high conservation scores in ConSurf output were assessed for solvent accessibility, stability, and secondary structure prediction by NetsurfP-2.0. The results are displayed in **Table 3**.

**Table 3.** NetsurfP-2.0 prediction based on relative solvent accessibility, stability, and secondary structure prediction

**Amino acid change**	**NetsurfP-2.0**					

	**Class assignment**	**RSA**	**ASA**	**Secondary structure**	**Phi**	**Psi**
V507F	Buried	13%	20 Å	α helix	−64°	−44°
R335W	Exposed	44%	101 Å	Coil	−110°	137°
E415K	Exposed	52%	90 Å	Strand/β sheet	−108°	137°
K591M	Exposed	37%	77 Å	α helix	−57°	−41°
E594K	Buried	6%	10 Å	α helix	−66°	−41°
F561Y	Buried	4%	8 Å	α helix	−60°	−38°
R609S	Buried	9%	22 Å	Strand/β sheet	−112°	132°

ASA, absolute surface accessibility; RSA, Relative surface accessibility.

### Analysis of structural impacts of high-risk nsSNPs in *STAT3*

**Figure 2.**
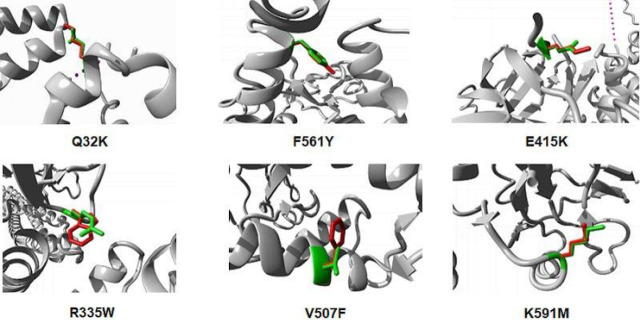
Structural variation of the wild-type and mutant residues by Project HOPE. The wild-type residue is presented as green and the mutant residue is shown in red.

HOPE showed the difference between the wild-type and mutant amino acids in connection with their physical and chemical properties, hydrophobicity, spatial structure, and function. Project HOPE server revealed that the mutant residues V507F, R335W, E415K, F561Y, and Q32K were bigger than the wild-type residues whereas the mutant residue of K591M was smaller than the wild-type. In addition, the mutant residue of R335W, K591M, and F561Y is more hydrophobic than the wild-type. The change in the size and hydrophobicity of the mutant residue can disrupt the H-bonding interaction with neighboring molecules. Moreover, the mutation at 335, 591 positions the wild-type residue charge was lost, whereas at 415^th^ position the mutant introduced an opposite charge; further, the mutant also introduced a charge at position 32. The variation in charge can cause a loss of interaction with other molecules. **Figure 2** illustrates the structural images of 6 deleterious nsSNPs showing both wild-type and mutant residues at the specific protein site.

### Secondary structure prediction and protein–protein interaction analysis

**Figure 3.**
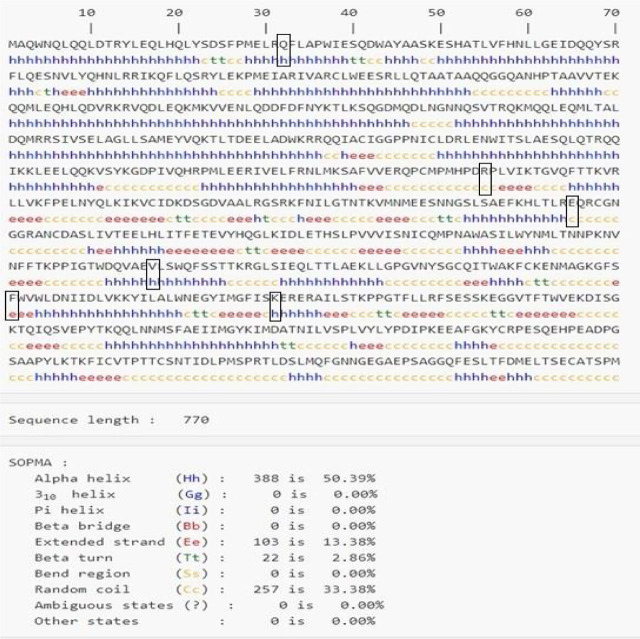
Secondary structure prediction and calculations using SOPMA. The SOPMA program predicts the secondary structure of the STAT3 protein. The black boxes represent wild amino acids that might be altered by STAT3 pathogenic nsSNPs. Alpha helix, extended strand, beta turn, and random coil are all represented by the letters “h,” “e,” “t,” and “c,” respectively. nsSNPs, non-synonymous single nucleotide polymorphisms; STAT, signal transducer and activator of transcription factors.

**Figure 4.**
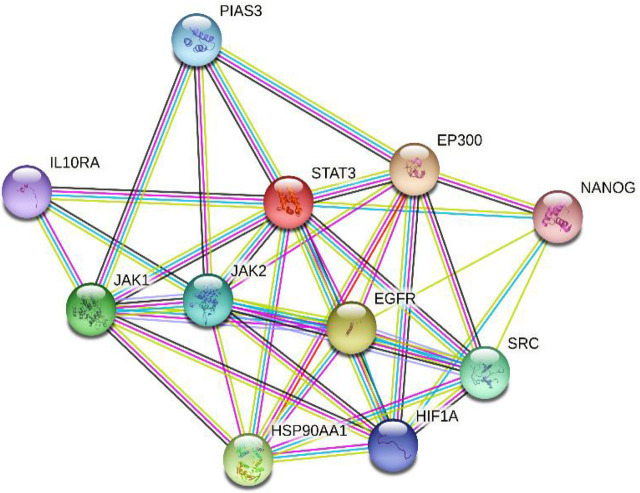
Protein–protein interaction network of *STAT3* using STRING server showing strong functional association with EP300, PIAS3, IL10RA, JAK1, JAK2, EGFR, HSP90AA1, SRC, and homeobox protein NANOG. A weak interaction has been observed for HIF1A. EGFR, epidermal growth factor receptor; EP300, histone acetyltransferase p300; HIF1A, hypoxia-inducible factor 1-alpha; HSP90AA1, heat shock protein HSP 90-alpha; IL10RA, interleukin-10 receptor; JAK, Janus kinases; PIAS3, E3 SUMO-protein ligase; SRC, proto-oncogene tyrosine-protein kinase; STAT, signal transducer and activator of transcription factors; STRING, Search Tool for the Retrieval of Interacting Genes/Proteins.

SOPMA predicted the secondary structure of STAT3, which explained the distributions of alpha-helix, beta-sheet, and random coil. The secondary structure prediction of STAT3 by SOPMA is shown in **Figure 3**. SOPMA secondary structure elements calculations showed that 50.39% of sites were in alpha helixes, 33.38% in random coils, 2.86% in beta twists, and 13.38% in extended strands. Out of the 6 high-risk nsSNPs, 2 were found in random coils, 3 in alpha helixes, and 1 in extended strands. The STRING maps were used to depict the protein–protein interaction of STAT3. The STRING results showed the functional interaction pattern of the STAT3 protein with other proteins in a cell, which is useful in interpreting the genotype–phenotype consequences of mutations. Histone acetyltransferase p300 (EP300), E3 SUMO-protein ligase (PIAS3), interleukin-10 receptor (IL10RA), JAK1, JAK2, epidermal growth factor receptor (EGFR), heat shock protein HSP 90-alpha (HSP90AA1), proto-oncogene tyrosine-protein kinase (SRC), and homeobox protein NANOG were all found to have substantial functional connections with STAT3 in the STRING prediction (**Figure 4**).

### Predicting ligand binding site effects of deleterious nsSNPS

**Figure 5.**
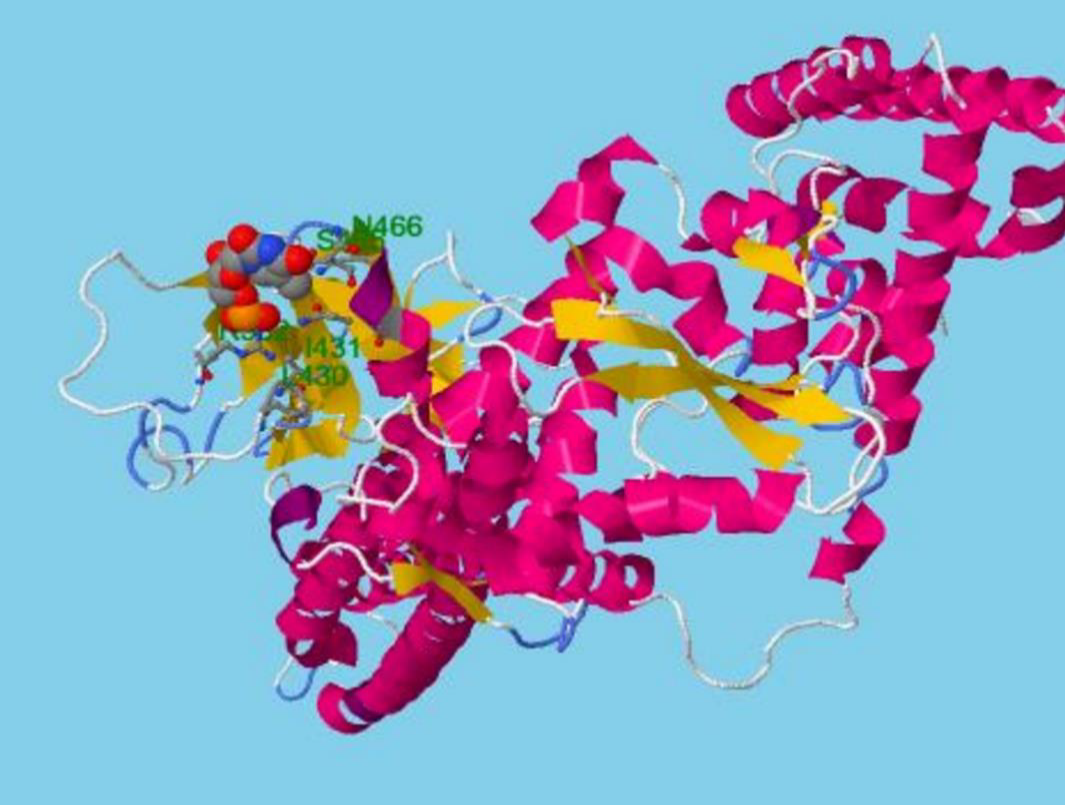
3D model and binding site residues for domain 1 predicted by RaptorX.

STAT3 ligand binding sites were predicted using the RaptorX Binding server. A pocket multiplicity number greater than 40, according to the RaptorX Binding server, suggests an accurate prediction. In the STAT3 protein, there were only 1 processed domain and 2 predicted pockets, one with a multiplicity of 11 that binds to R382 L430 I431 S465 N466 Q469 and the other with a multiplicity of 3 that binds to M331 H332 I467. The server predicted that 128 locations were disordered. RaptorX also predicted the secondary structure showing 45% helix, 13% sheets, and 41% coil. **Figure 5** shows the structure predicted by the RaptorX Binding server.

### Gene–gene interactions

**Figure 6.**
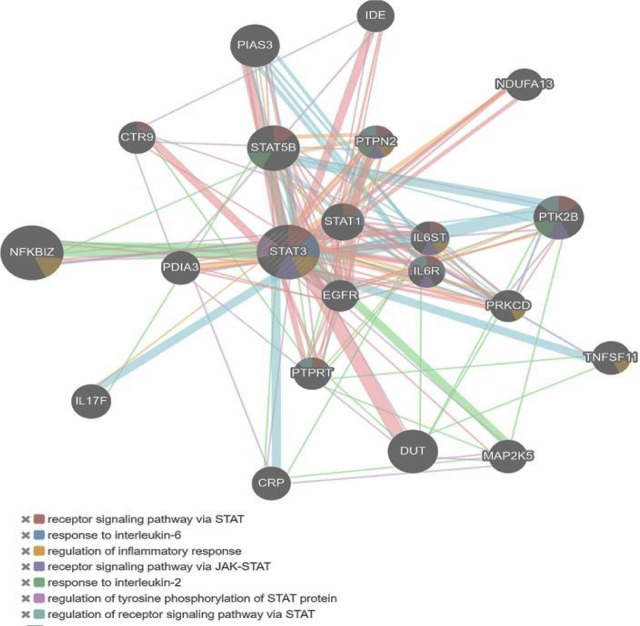
Gene–gene interaction network of *STAT3* gene shows physical and genetic interaction with nuclear factor kappa B subunit 1 (*NFKB1Z*) and mitogen-activated protein kinase kinase 5 (*MAP2K5*), *EGFR*, and signal transducer and activator of transcription 1 (*STAT1*). EGFR, epidermal growth factor receptor; STAT, signal transducer and activator of transcription factors.

GeneMANIA server predicted the functional gene–gene interaction network of *STAT3*, which is shown in **Figure 6**. According to the predicted results, *STAT3* interacts physically and genetically with nuclear factor kappa B Subunit 1 (*NFKB1Z*) and mitogen-activated protein kinase kinase 5 (*MAP2K5*), *EGFR,* and *STAT1*, and is co-expressed mostly with *EGFR*, protein disulfide-isomerase A3 (*PD1A3*), interleukin 6 receptor (*IL6R*), and *STAT5B*.

### 3D structure of *STAT3* protein

**Figure 7.**
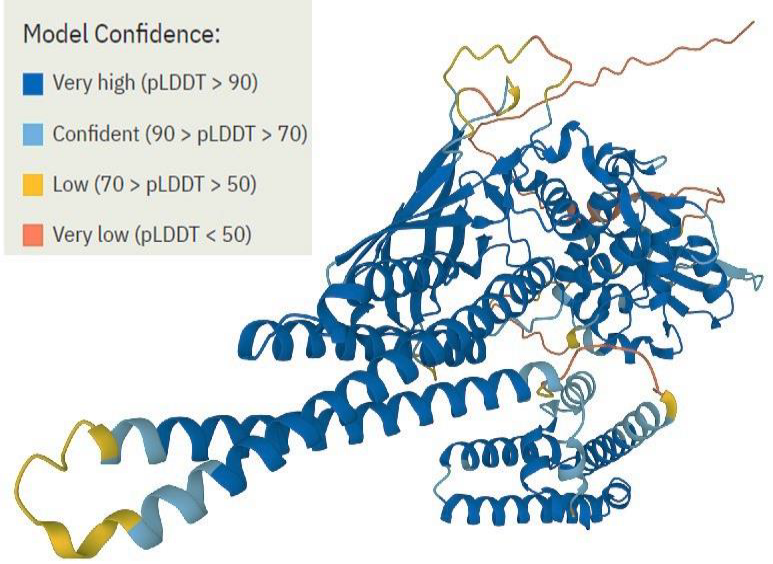
3D structure prediction of STAT3 by AlphaFold. STAT, signal transducer and activator of transcription factors.

The overall confidence for the whole protein chain is shown by the average pLDDT scores across all residues The Alpha-Fold algorithm produces a pLDDT score for each individual residue that ranges from 0 to 100. In STAT3 3D structure, a very high degree of confidence (pLDDT >90) was obtained and majority of the 3D structural area belongs to α-helical domains (**Figure 7**).

### Protein stability correlation upon amino acid substitution

**Figure 8.**
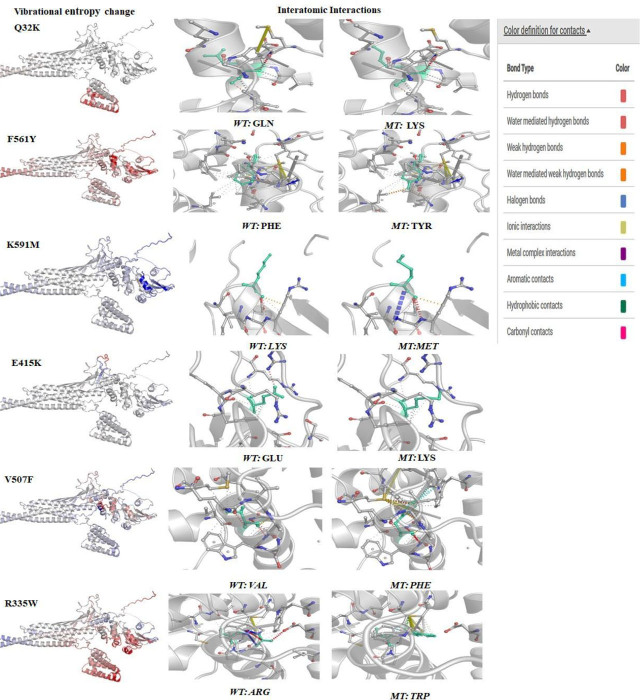
Visual representation of protein flexible conformation based on the vibrational entropy difference (ΔΔS) and the interatomic interaction between wild-type and mutant structures on STAT3 structure. Amino acids colored according to the vibrational entropy change upon mutation. BLUE represents a rigidification of the structure and RED a gain in flexibility. WT and MT residues are depicted as light-green sticks alongside the surrounding residues that are involved in any form of interaction.

DynaMut calculates the effect of point mutations on protein stability and flexibility based on interatomic interactions, as summarized in **Table 4**. DynaMut identified the ∆∆G prediction of F561Y as destabilizing; however, NMA-based predictions revealed that all 6 deleterious nsSNPs reduce structural stability when compared to the wild-type protein. **Figure 8** depicts the difference in vibrational entropy and interatomic interactions between the wild-type and mutant.

**Table 4.** Prediction of protein stability using DynaMut server

**Amino acid change**	**Stability-based prediction**					**Δ Vibrational entropy energy**	

	**ΔΔG kcal/mol**	**NMA-based predictions ΔΔG ENCoM**	**Other structure-based predictions**			**ΔΔS_Vib_ ENCoM (kcal/mol/K)**	**Flexibility**

			**ΔΔG mCSM (kcal/mol)**	**DDG SDM (kcal/mol)**	**ΔΔG DUET (kcal/mol)**		
Q32K	0.087 (Stabilizing)	−0.009 kcal/mol (Destabilizing)	−0.453 (Destabilizing)	0.030 (Stabilizing)	−0.019 (Destabilizing)	0.012	Increase of molecule flexibility
F561Y	−0.705 (Destabilizing)	−0.089 (Destabilizing)	−0.673 (Destabilizing)	−1.030 (Destabilizing)	−0.560 (Destabilizing)	0.111	Increase of molecule flexibility
K591M	0.057 (Stabilizing)	0.016 (Destabilizing)	0.335 (Stabilizing)	0.130 (Stabilizing)	0.485 (Stabilizing)	−0.019	Decrease of molecule flexibility
E415K	0.107 (Stabilizing)	0.023 (Destabilizing)	−0.461 (Destabilizing)	−0.150 (Destabilizing)	−0.062 (Destabilizing)	−0.029	Decrease of molecule flexibility
V507F	0.627 (Stabilizing)	0.195 (Destabilizing)	−1.244 (Destabilizing)	−1.190 (Destabilizing)	1.458 (Destabilizing)	−0.244	Decrease of molecule flexibility
R335W	0.050 (Stabilizing)	−0.172 (Destabilizing)	−0.198 (Destabilizing)	−0.010 (Destabilizing)	−0.447 (Destabilizing)	0.215	Increase of molecule flexibility

ENCoM, elastic network contact model; NMA, normal mode analysis.

## Discussion

In mammals, several STAT proteins play an important role in host defense. Despite its importance, STAT3 is linked to the JAK family of proteins and is capable of integrating signals from several signaling pathways. STAT3 that is persistently active has been associated with various malignancies, though it is most commonly associated with head and neck cancer and multiple myelomas [1]. The occurrence of deleterious SNPs in various disease-related genes has made in silico analysis of deleterious SNPs from large databases a major concern in recent years [28]. Prioritization of the most deleterious SNPs enables their use as markers in genetic disease screening and helps in the formulation of personalized treatment strategies. Studying the structure and function of the *STAT3* using a variety of computational methods determines the effects of deleterious nsSNPs in *STAT3* and their association with various diseases.

In this study, by merging numerous in silico-based SNP prediction algorithms, we projected 6 deleterious SNPs that are considered high-risk and valid, and they were further examined. The nsSNPsrs145786768, rs193922716, rs193922717, rs193922719, rs1064116, and rs180312 with amino acid changes V507F, R335W, E415K, K591M, F561Y, and Q32K were considered deleterious using 6 in silico SNP prediction tools SIFT, Polyphen-2, PANTHER, SNP&GO, PROVEAN, and PHD -SNP. Based on the conservation profile, structural conformation, relative solvent accessibility, secondary structure prediction, and protein–protein interaction, all of the 6 nsSNPs were identified as the most deleterious nsSNPs. Protein stability is essential for a protein's structural and functional activity. The I-Mutant tool revealed that the predicted 6 nsSNPs on the STAT3 protein had decreased stability. Protein stability determines the conformational structure and function of the protein. Misfolding, degradation, or abnormal protein conglomeration can be affected by changes in protein stability [29].

Conserved residues of the protein were involved in biological system management, including stability, folding, or both of these. Functional amino acids that are present in enzymatic sites interact with the proteins in a significant manner and when compared to other residues in the protein, these residues are found to be more conserved [30]. The evolutionary conservation profile of STAT3 protein by ConSurf revealed that the 570, 335, 415, 591, 561, and 32 amino acid positions were located in highly conserved regions and the 507 position was predicted to be structural and buried whereas the rest of the positions were functional and exposed. Combining the result of both ConSurf and I-Mutant, the predicted 6 nsSNPs are potentially high-risk variants due to their ability to decrease the protein stability and higher conservancy. The Project HOPE results indicate that the wild-type residue Valine at position 507 and Phenylalanine at position 561 are highly conserved and that neither the mutant nor another residue with similar properties could be observed in other homologous sequences, which justifies the inference that the mutations are probably damaging to the protein.

The exposed variants were located on the surface of the protein, which might lead to loss of interactions and structural alterations, particularly in the transmembrane domains. Repulsion, misfolding, and loss of interactions may occur from the addition or removal of charge or hydrophobicity. SOPMA secondary structure calculations showed that the deleterious nsSNPs are mainly found in helix and coil regions rather than β turns. RaptorX predicted the high-risk nsSNPs as ligand binding locations.

Protein–protein interaction network is an important factor in understanding biological processes. Using STRING, the functional genomics data and structural assessment, functional, and evolutionary features of STAT3 protein were analyzed [31]. EP300, PIAS3, IL10RA, JAK1, JAK2, EGFR, HSP90AA1, SRC, and NANOG were found to have strong functional association with STAT3 protein. Amino acid change can alter the structure of a protein, and consequently its function. As a result, the variant protein with deleterious SNPs may interact with other proteins, and thereby cause phenotypic changes and protein expression [32]. The structural domains of STAT3 involved in protein–protein interactions allow selective inhibition of a group of *STAT3*-targeted genes associated with oncogenesis to minimize therapeutic toxicity [33]. Based on analysis of interaction patterns and co-expression profiles, we may infer that the harmful nsSNPs in the *STAT3* gene might influence and disrupt the proper functioning of related genes, which highlights the importance of these linked and co-expressed genes in immune pathways and inflammation-associated tumorigenesis. The need for the acquisition of a higher quality of targeted protein structure and the validation of experimental models in turn necessitate further investigation into analysis of the nsSNPs-induced deteriorative changes in STAT3. In AlphaFold, pLDDT computes the degree of prediction and experimental structure of STAT3. The DynaMut tool provides both the stability change and the difference in entropy energy between wild-type and mutant structures. The 6 nsSNPs were found to affect the molecular flexibility of the STAT3 protein by altering its structural conformation.

Deleterious SNPs of the *STAT3* gene are capable of regulating the expression, stimulation, and predisposition in inflammatory and neoplastic diseases [34]. *STAT3* activation was detected in various tumors in association with the proliferation, invasion, and angiogenesis of malignant cells and the inhibition of anti-tumor immunity [35]. Although clinical studies are more consistent in identifying harmful nsSNPs, it takes a longer period to conduct regular experiments on all variants, and different techniques have various levels of reliability for threat prediction. The methods used in this study provide evidence of the many effects of mutations, making it easier to identify pathogenicity [36]. The study does, however, have some limitations. The number of reported causative nsSNPs was limited and clinical analysis and experiments are required to confirm the effects of these nsSNPs.

## Conclusions

The transcription factor *STAT3* exerts important effects on tumorigenesis and tumor-related inflammations. Variations in *STAT3* are associated with various human tumors. The present study screened 417 nsSNPs of the *STAT3* gene using different computational tools and scrutinized 6 nsSNPs with amino acid changes, namely V507F, R335W, E415K, K591M, F561Y, and Q32K, as most deleterious. The deleterious variants may affect structural and cellular function. *STAT3* is involved in different tumors and the identified deleterious nsSNPs from our study could be important candidates and could be used as diagnostic markers. Based on our result, we can conclude that these 6 nsSNPs should be considered as potential biomarkers in causing diseases related to *STAT3* variations. However, in silico tools cannot replace conclusive trials, and their conclusions should be double-checked by additional biological evaluation.

## References

[1] Levy DE, Darnell JE (2002). Stats: transcriptional control and biological impact. Nat Rev Mol Cell Biol..

[2] Villarino AV, Kanno Y, Ferdinand JR, O’Shea JJ (2015). Mechanisms of Jak/STAT signaling in immunity and disease. J Immunol..

[3] Buettner R, Mora LB, Jove R (2002). Activated STAT signaling in human tumors provides novel molecular targets for therapeutic intervention. Clin Cancer Res..

[4] Colomiere M, Ward AC, Riley C, Trenerry MK, Cameron-Smith D, Findlay J (2009). Cross talk of signals between EGFR and IL-6R through JAK2/STAT3 mediate epithelial-mesenchymal transition in ovarian carcinomas. Br J Cancer..

[5] Pawlus MR, Wang L, Hu CJ (2014). STAT3 and HIF1α cooperatively activate HIF1 target genes in MDA-MB-231 and RCC4 cells. Oncogene..

[6] Kotkowska A, Sewerynek E, Domańska D, Pastuszak-Lewandoska D, Brzeziańska E (2015). Single nucleotide polymorphisms in the STAT3 gene influence AITD susceptibility, thyroid autoantibody levels, and IL6 and IL17 secretion. Cell Mol Biol Lett..

[7] Yuan K, Liu H, Huang L, Ren X, Liu J, Dong X (2014). rs744166 polymorphism of the STAT3 gene is associated with risk of gastric cancer in a Chinese population. Biomed Res Int..

[8] Collins FS, Brooks LD, Chakravarti A (1998). A DNA polymorphism discovery resource for research on human genetic variation. Genome Res..

[9] Goswami AM (2015). Structural modeling and in silico analysis of non-synonymous single nucleotide polymorphisms of human 3β-hydroxysteroid dehydrogenase type 2. Meta Gene..

[10] Sim N-L, Kumar P, Hu J, Henikoff S, Schneider G, Ng PC (2012). SIFT web server: predicting effects of amino acid substitutions on proteins. Nucleic Acids Res..

[11] Adzhubei IA, Schmidt S, Peshkin L, Ramensky VE, Gerasimova A, Bork P (2010). A method and server for predicting damaging missense mutations. Nat Methods..

[12] Choi Y, Sims GE, Murphy S, Miller JR, Chan AP (2012). Predicting the functional effect of amino acid substitutions and indels. PLoS One..

[13] Thomas PD, Kejariwal A, Guo N, Mi H, Campbell MJ, Muruganujan A, Lazareva-Ulitsky B (2006). Applications for protein sequence–function evolution data: mRNA/protein expression analysis and coding SNP scoring tools. Nucleic Acids Res..

[14] Calabrese R, Capriotti E, Fariselli P, Martelli PL, Casadio R (2009). Functional annotations improve the predictive score of human disease-related mutations in proteins. Hum Mutat..

[15] Capriotti E, Calabrese R, Casadio R (2006). Predicting the insurgence of human genetic diseases associated to single point protein mutations with support vector machines and evolutionary information. Bioinformatics..

[16] Capriotti E, Fariselli P, Casadio R (2005). I-Mutant2. 0: predicting stability changes upon mutation from the protein sequence or structure. Nucleic Acids Res..

[17] Elkhattabi L, Morjane I, Charoute H, Amghar S, Bouafi H, Elkarhat Z (2019). In silico analysis of coding/noncoding SNPs of human RETN gene and characterization of their impact on resistin stability and structure. J Diabetes Res..

[18] Ashkenazy H, Abadi S, Martz E, Chay O, Mayrose I, Pupko T, Ben-Tal N (2016). ConSurf 2016: an improved methodology to estimate and visualize evolutionary conservation in macromolecules. Nucleic Acids Res..

[19] Petersen B, Petersen TN, Andersen P, Nielsen M, Lundegaard C (2009). A generic method for assignment of reliability scores applied to solvent accessibility predictions. BMC Struct Biol..

[20] Venselaar H, Te Beek TA, Kuipers RK, Hekkelman ML, Vriend G (2010). Protein structure analysis of mutations causing inheritable diseases. An e-Science approach with life scientist friendly interfaces. BMC Bioinformatics..

[21] Geourjon C, Deléage G (1995). SOPMA: significant improvements in protein secondary structure prediction by consensus prediction from multiple alignments. Comput Appl Biosci..

[22] Warde-Farley D, Donaldson SL, Comes O, Zuberi K, Badrawi R, Chao P (2010). The gene MANIA prediction server: biological network integration for gene prioritization and predicting gene function. Nucleic Acids Res..

[23] Jumper J, Evans R, Pritzel A, Green T, Figurnov M, Ronneberger O (2021). Highly accurate protein structure prediction with AlphaFold. Nature..

[24] Mariani V, Biasini M, Barbato A, Schwede T (2013). lDDT: a local superposition-free score for comparing protein structures and models using distance difference tests. Bioinformatics..

[25] Pires DE, Ascher DB, Blundell TL (2014). mCSM: predicting the effects of mutations in proteins using graph-based signatures. Bioinformatics..

[26] Worth CL, Preissner R, Blundell TL (2011). SDM—a server for predicting effects of mutations on protein stability and malfunction. Nucleic Acids Res..

[27] Pires DE, Ascher DB, Blundell TL (2014). DUET: a server for predicting effects of mutations on protein stability using an integrated computational approach. Nucleic Acids Res..

[28] Rozario LT, Sharker T, Nila TA (2021). In silico analysis of deleterious SNPs of human MTUS1 gene and their impacts on subsequent protein structure and function. PLoS One..

[29] Witham S, Takano K, Schwartz C, Alexov E (2011). A missense mutation in CLIC2 associated with intellectual disability is predicted by in silico modeling to affect protein stability and dynamics. Proteins Struct Funct Bioinforma..

[30] Islam MJ, Khan AM, Parves MR, Hossain MN, Halim MA (2019). Prediction of deleterious non-synonymous SNPs of human STK11 gene by combining algorithms, molecular docking, and molecular dynamics simulation. Sci Rep..

[31] Jensen LJ, Kuhn M, Stark M, Chaffron S, Creevey C, Muller J (2009). STRING 8—a global view on proteins and their functional interactions in 630 organisms. Nucleic Acids Res..

[32] Mohamoud HS, Hussain MR, El-Harouni AA, Shaik NA, Qasmi ZU, Merican AF (2014). First comprehensive in silico analysis of the functional and structural consequences of SNPs in human GalNAc-T1 gene. Comput Math Methods Med..

[33] Yeh JE, Frank DA (2016). STAT3-interacting proteins as modulators of transcription factor function: implications to targeted cancer therapy. Chem Med Chem..

[34] Yan R, Lin F, Hu C, Tong S (2015). Association between STAT3 polymorphisms and cancer risk: a meta-analysis. Mol Genet genomics..

[35] Yu H, Lee H, Herrmann A, Buettner R, Jove R (2014). Revisiting STAT3 signalling in cancer: new and unexpected biological functions. Nat Rev Cancer..

[36] Mustafa MI, Murshed NS, Abdelmoneim AH, Makhawi AM (2020). In silico analysis of the functional and structural consequences of SNPs in human ARX gene associated with EIEE1. Informatics Med Unlocked..

